# Progress in conducting and reporting behaviour change intervention studies: a prospective retrospection

**DOI:** 10.1080/21642850.2021.1939701

**Published:** 2021-06-21

**Authors:** Marie Johnston

**Affiliations:** Aberdeen Health Psychology Group, Institute of Applied Health Sciences, Aberdeen, UK

**Keywords:** Behaviour change, trials, evidence synthesis, review, reporting

## Abstract

**Background:**

ehaviour change is a key to addressing many health and healthcare problems and interventions have been designed to improve health outcomes. These behaviour change interventions have been evaluated in many ways, including randomised controlled trials, and over recent decades there has been considerable progress in the conduct and reporting these studies. This paper is a personal retrospection on the changes occurring that have resulted in our current improved methods and their potential for future advancement.

**Advances:**

There has been steady development of methods for conducting trials, including advances in statistical methods enabled by increase computing power and programmes, greater attention to the recruitment of participants and in the specification of outcomes. Trial reporting has improved, largely due to publication of guidelines for reporting interventions and trials, but until recently the reporting of behaviour change interventions has been quite limited. Developments in the specification of active ingredients of these interventions, the behaviour change techniques, has transformed our ability to report interventions in a manner that facilitates evidence synthesis and enables replication and implementation. However, further work using ontological approaches is needed to adequately represent the evidence contained in the mass of accumulated studies. Meanwhile, attention is gradually being paid to the comparator groups in trials leading to better reporting but with continuing challenges about how control groups are selected.

**Conclusions:**

These developments are important for the advancements of behavioural science – but also in consolidating the expertise needed to address global social, environmental and health challenges.

Behaviour and behaviour change are key to addressing many health, social and environmental challenges. Behaviours influence both mortality and disability, globally (Mokdad, Marks, Stroup, & Gerberding, 2004; Loprinzi, 2016; WHO, 2009). They are integral to the delivery of healthcare and in patients’ responses to healthcare, and behavioural problems may be the target for healthcare interventions. Behaviour change interventions (BCIs) have been implemented by diverse agencies and practitioners, such as educators, legislators, advertisers as well as behavioural scientists. There is no agreed standard of how we report BCIs and their evaluation, but guidelines have resulted in considerable improvement over recent decades. Here I present a personal account of what has been learned and what it would have been good to know years ago to improve the scientific and practical value of BCI research conducted.

Historically, the style and quality of BCI reporting adopted to ensure that BCIs are replicated faithfully has differed for different types of BCI. Laboratory studies have adopted the standards of science, i.e. enough detail of what was actually done, and what was delivered, for other scientists to replicate the methods. By contrast, field studies in which BCIs are delivered as clinical or healthcare interventions may give quite limited descriptions of the intervention and therefore present challenges for those wishing to replicate, or for systematic reviewers trying to decipher what BCI content was actually delivered. Three papers we published in the 1970s illustrate this problem. The laboratory study (Johnston, 1976) describes a series of experiments comparing the learning of delinquents and non-delinquents under conditions of social or financial reward in tasks involving either tracking of light or construction of sentences. The report details participant selection, recruitment and instructions for the task; precise information about the context, equipment and spatial layout of the room are provided and every word of the BCI active content is reported. The clinical study (Johnston, 1977) describes the behavioural treatment for agoraphobia, for a series of patients seen in a primary care setting. The paper refers to a manual for detail of the BCI. Unlike the laboratory study, further safeguarding of reliable implementation of the BCIs clinically was provided by extensive supervision arrangements. The healthcare intervention (Johnston & Lee-Jones, 1979) describes a randomised controlled trial of the management of post-surgical patients in either a district general or community hospital, investigating return to usual activities of daily living, but little information is given about the intervention other than referring to a policy paper describing the development of community hospitals. The community hospital intervention aimed to achieve earlier discharge from the district general hospital in order to reduce costs, limit exposure to nosocomial infection and speed return to usual environment and activities. The community hospital was implemented in two settings, each being compared with the same district general hospital; the results showed that one community hospital had superior results, the other inferior results to the district general hospital, reflecting differences in the implementation of what was purported to be the same BCI. Clearly, the reporting of the healthcare BCI leaves considerable scope for improvement over subsequent years.

Good quality reporting is important for several reasons. First, if a successful BCI is poorly reported it will be difficult to implement it reliably in practice, resulting in poor outcomes for individuals who might have benefited from the successful BCI. Second, practitioners may wrongly believe they are implementing the successful BCI when they are omitting essential ingredients. Third, unsuccessful or even harmful interventions may be repeated, resulting in both potential harm and waste of resources. Fourth, if BCIs are poorly reported, evidence syntheses may not be able to incorporate evidence from the poorly reported studies, or it may involve writing to authors for information about the BCI content, a procedure that only succeeds if the details have been stored. Fifth, the science of behaviour change will advance more slowly if the best evidence is unusable as BCIs are not adequately reported: every science depends on good reporting of methods, analyses and results in order to progress.

The quality of reporting of BCI studies must be evaluated against two separate sets of criteria: the criteria for conducting and reporting the methods of testing the success of the BCI and the standards for reporting the intervention itself. Since the 1970s progress has been made, some due to advances in computing technology, but much of which could technically have been included in the 1970s if the guidance had been available.

## Conducting and reporting BCI trials

While *randomised controlled trials* (RCTs) had been used in other disciplines and in evaluating the effectiveness of drugs, it was only in the 1970s that they were applied in studies of healthcare interventions. Early studies, such as Johnston and Lee-Jones (1979), reported methods of randomisation of participants to conditions but were frequently weak on detail concerning other potential sources of bias. The 1996 publication of CONSORT guidelines http://www.consort-statement.org/ proposed the necessary information to be reported for a healthcare intervention and updating of these guidelines, with extensions such as ‘social and psychological interventions’ http://www.consort-statement.org/extensions/overview/social-and-psychological-interventions, have substantially improved reporting. The initial guideline was for parallel-group studies but later additions have benefited from developments in statistical and computing methods and provide guidelines for reporting other trial designs including cluster randomised and N-of-1 trial designs (Vieira, McDonald, Araújo-Soares, Sniehotta, & Henderson, 2017). In addition, there have been influential documents on the methods of development of complex interventions. The UK Medical Research Council’s first guidance document recommended a linear sequence in the development, paralleling the methods used in drug trials (Campbell et al., 2000). The next version emphasised a more iterative process and a greater variety of research designs (Craig et al., 2008). The most recent version will put more emphasis on the developments of complex interventions within their context and with more stress on a systems approach (Simpson & Moore, 2021). This would surely have made more sense of the results of the community hospital studies of Johnston & Lee-Jones as the results were probably mainly influenced by the historical context of the local hospitals and their status in their respective communities.

Meanwhile methods of *synthesising evidence* from RCTS was developing. Methods of conducting and reporting systematic reviews were advanced by the creation of the Cochrane Collaboration (Chalmers, Dickersin, & Chalmers, 1992; https://www.cochrane.org/ ) and the evaluation of bias in the RCTs included using the ‘risk-of-bias’ tool https://www.riskofbias.info/. Prior to the mid-1990s, reviewers had to find papers by hand-searching and correspondence with authors (e.g. Johnston & Vögele, 1993); the later Powell et al. (2016) updating of Johnston & Vogele’s study illustrates methods and reporting of systematic reviews following the development of Cochrane and PRISMA (Page et al., 2021) guidance. While some progress has depended on digital technologies, especially in finding relevant studies (e.g. Shemilt et al., 2014) many of these advances could have been implemented with the resources available at any time.

Technology has particularly advanced *statistical methods* that require vast amounts of computing power, such as multi-level modelling, time series and latent growth modelling but other progress in research designs and trial analysis could have been made without it. The ‘intention-to-treat’ principle was gradually introduced to overcome bias in the design, analysis and interpretation of trial results but even as late as the 1990s this principle was being applied and reported incorrectly (Hollis & Campbell, 1999), especially in dealing with participants allocated to an active treatment who, for various reasons, fail to receive the treatment. More recently other methods of conducting analyses have been used to allow interpretation and appropriate reporting of results when some participants allocated to a treatment do not receive it, including per-protocol participant allocation to intervention arms in pragmatic trials and methods of allowing for participant ‘compliance’ with the intervention protocol (Peugh, Strotman, McGrady, Rausch, & Kashikar-Zuck, 2017). Zelen methods were designed to incorporate consent procedures into the design and therefore more precise reporting of randomisation procedures (Bradley, 1993).

Despite *factorial designs* being frequently used in laboratory experiments they are not used frequently in trials of health care interventions. This is surprising given the gain in power for testing more than one intervention simultaneously and the obvious importance of evaluating the joint effect of two interventions. For example, in a test of an educational intervention and a reward-based intervention using a factorial design to test single and combined effects, we found the rewards were effective, but the educational intervention was not, and the educational intervention did not increase the improvement in dentists’ clinical management of children’s teeth (Clarkson et al., 2008). Some combinations of interventions may be either impossible or improbable and Collins, Murphy, and Strecher (2007) have proposed that fractional factorial designs could be used to test the most probable combinations of intervention components as part of the MOST methods.

More explicit reporting of the rationale for *numbers of participants and methods of recruitment* have also been developed resulting in trials that have the power to examine the research question. Prior to standardised methods of conducting power calculations, authors typically provided no explanation of the number of participants included and may have been conducting underpowered trials. For example, I was involved in an early trial of therapeutic communities to reduce delinquent recidivism conducted before the days of power-analyses. My main contribution was to calculate that, given the maximum possible rate of throughput, it would be impossible to achieve a significant effect in less than 10 years; as a result the trial was stopped and not reported. At a later date, this calculation would have been done in advance avoiding much inconvenience, waste of resources and, potentially, reporting of an under-powered trial. Methods of conducting power calculations are now available for many different research designs (Perugini, Gallucci, & Costantini, 2018). In addition, more care is being paid to the recruitment of participants that represent the target population without the selection bias that may result from convenience samples and greater attention is paid to factors that may bias recruitment and retention in trials (Bricca et al., 2021).

The reason for selecting and scheduling *outcome variables* has also improved. In early healthcare evaluation studies, outcomes were rarely based on a model of the processes involved and measures were a mixture of clinical, behavioural and social outcomes (McDowell, 2006). Outcomes of BCI trials may be specified as behaviour or, based on the model, the resulting health outcome. The WHO ICF model identifies potential outcomes as impairment, disability (limitations) or participation (restrictions) and different BCIs may impact different outcomes (WHO). For example, a surgical intervention might address impairment, a physiotherapy intervention activity limitations and a social or psychological intervention might increase social participation. However, outcome measures frequently represent a mixture of these three outcomes (Pollard, Johnston, & Dieppe, 2006) and may be insensitive to the actual mode of action of the interventions. Even when the target outcome is clear it is also important to specify when that outcome should be assessed, and recent trials have been more precise in specifying the pattern of improvement and relapse over time. For BCIs targeting a habitual health-related behaviour, time is required for a change of habits to be observed, and for relapsing behaviours such as smoking cessation, at least a year is usually scheduled (Black, Johnston et al., 2020; Black et al., 2020). Where the BCI targets a recovery process, then evidence about usual recovery times is valuable in specifying the outcome measurement date; for example, recovery from stroke reaches a plateau at about 6 months and trials typically make assessments at that point (Johnston et al., 2007). Intensive longitudinal methods, using ecological momentary assessment (EMA) also allow the assessment of progress toward the target outcome. Berli, Inauen, Stadler, Scholz, and Shrout (2020) discuss methods of distinguishing when an endpoint outcome may be the result of a steady, improving or relapsing pattern.

Clearly, there is much in current knowledge and practice in the conduct and reporting of BCI evaluations and more generally in trials and evidence synthesis that it would have been advantageous to know about in earlier years. While some of these improvements depend on technological developments, most of the things mentioned here could have been implemented 30 or 40 years ago but it has taken these years to clarify and standardise improved practices. Can the same be said about how we conduct and report the interventions themselves?

## Conducting and reporting behaviour change interventions

As the paper on community hospitals illustrates, early *reporting of complex interventions* was very limited and not adequate for ensuring replication (Johnston & Lee-Jones); the two implementations of the intervention resulted in opposite results clearly indicating that something different was happening in the two community hospitals. While reporting of trial methodology improved in the late twentieth century, well-designed, expensive trials were still being used to evaluate BCI interventions which were scantily described and based on little more than the authors’ ideas of how behaviour might be changed (Johnston, 1995). Even when we attempted to report BCIs well, it was not clear how it should be done. In the mid-1990s, a journal editor encouraged our research team to write *more* to describe the content of a cardiac rehabilitation BCI, but we had only vague ideas of how to do this. As a result, despite this generous invitation, the resulting description is very clear on when, where, how and by whom the intervention was delivered, and indicated how it was tailored to individuals but the complete description of the *content* gave remarkably little guidance:

The counselor provided information, guided action plans, gave advice, and provided leaflets and videos that were already available, i.e. they were not produced for this program. The patients were encouraged to take control by contributing to their own assessment and plans. For example, the choice of topic was guided by the patient’s choice from the following written menu. (Johnston, Foulkes, Johnston, Pollard, & Gudmundsdottir, 1999)

Reference was made to a manual which, at that pre-digital time, would have been unavailable to researchers or practitioners reading the paper and so, the net result was a very successful intervention that could not be reliably replicated. Anecdotally, we believe that some practitioners may have replicated the methods of delivery but may not have managed to replicate the *active content* of the BCI.

The first attempt to create standards of reporting led to the publication of the first CONSORT (Consolidated Standards of Reporting Trials) statement (Begg et al., 1996) but the only guidance on reporting the intervention was the item: ‘Planned interventions and their timing’. The updated guidance in 2001 improved many aspects of reporting, but when it came to reporting the intervention it only specified: ‘ *Item 4.* Precise details of the interventions intended for each group and how and when they were actually administered … Authors should describe each intervention thoroughly, including control interventions’ (Moher et al., 2001). In 2020, Davidson et al., discussed the requirements of BCIs and recommended CONSORT guidelines for reporting with some additional guidance on describing the intervention and at the same time supported use of Cochrane standards for systematic reviews.

However, it became clear that more was needed and the TIDieR checklist (Template for Intervention description and replication) (Hoffmann et al., 2014) recognised the need for reporting that enabled replication of the intervention, for any type of intervention; more recently variations have been developed for public health interventions (Campbell et al., 2018; Cotterill, John, & Johnston, 2020). However, there is ample evidence that the reporting of BCIs is inferior to the reporting of other interventions (Johnston, 2014). Nonpharmacological interventions are more poorly reported than pharmacological interventions (Hoffmann, Erueti, & Glasziou, 2013) and, compared with other non-pharmacological interventions, BCI reports give less information about the BCI in the titles and abstracts of their papers (McCleary, Duncan, Stewart, & Francis, 2013). While TIDieR clarifies the essentials that should be reported, the template is generic and applies equally well to the reporting of surgical, rehabilitation and psychological interventions rather than being adapted for BCIs. Further details have been specified for social and psychological trials (Montgomery et al., 2018) in their extension to CONSORT, they specify Item 5 as ‘The interventions for each group with sufficient details to allow replication, including how and when they were actually administered^’^ and provide additional detail:

5a: Extent to which interventions were actually delivered by providers and taken up by participants as planned; 5b: Where other informational materials about delivering the intervention can be accessed: 5c: When applicable, how intervention providers were assigned to each group.

Even this update did not advance the description of active BCI content.

The most significant advance in reporting BCIs came with Abraham and Michie’s (2008) introduction of the term *‘behaviour change techniques’* (BCTs), now a term defined in the Encyclopedia of Behavioral Medicine as in the text box. In an earlier paper we had developed: ‘ a taxonomy of behaviour change programmes … for classification of underlying model, behaviour change methods, and modes of delivery’ (Hardeman, Griffin, Johnston, Kinmonth, & Wareham, 2000) but this was extremely limited compared with the subsequent work by Michie and colleagues leading to the development of several domain specific taxonomies and eventually to the generic Behaviour Change Technique Taxonomy v1 (BCTTv1) of 93-item hierarchically organised BCTs (Michie et al., 2013),which brought together many of the taxonomies that had developed in the interim, and evaluated the resulting taxonomy in a series of studies (Michie et al., 2015). BCTTv1 has been used and cited over 2500 times, especially as a means of synthesising evidence across BCI studies which used BCTTv1 to identify and therefore integrate findings over a varied range of BCTs. It has also been used to develop a tool linking BCTs to the theoretical mechanism of action based on triangulation of evidence from literature synthesis and expert consensus (Johnston et al., 2020).

**Table:** 

**“A behavior change technique (BCT)** is a systematic procedure included as an active component of an intervention designed to change behavior.The defining characteristics of a BCT are that it is: • A component of an intervention designed to change a specified behavior • The smallest (or smallest for the particular purpose) component that can be postulated to be an active ingredient within the intervention • An observable activity • Replicable • Specified by an active verb and clarity about the desired behavior change targeted with enough detail to achieve good agreement between experts A BCT is the smallest component of an intervention compatible with retaining the postulated active ingredients, and can be used alone or in combination with other BCTs." [Definition of Behaviour Change Technique (Michie, Johnston, & Carey, 2020)].

At the same time Kok and colleagues had been developing a classification of behaviour change methods, also linked to theory, within an Intervention Mapping approach to BCI development and evaluation (Kok et al., 2016). From a dearth of methods for reporting BCIs there is now a choice of systematic methods of describing interventions in a way that enables better replication than was possible even in the previous 20 years.

Despite these improvements in reporting BCIs, there continues to be poor reporting of the active content or the rationale for the *control or comparator groups* against which BCIs are evaluated. When we presented the research on evaluating early post-surgical discharge to community hospitals, Archie Cochrane (Winkelstein, 2009) was in the audience and he challenged me to explain why we had chosen to compare continuing district general hospital care with early discharge to community hospitals, suggesting that it might have been more appropriate to compare it with early discharge to home. As in so many things he anticipated subsequent developments, and here he illustrated the importance of appropriate and justified comparator selection.

A new intervention will look ‘better’ if compared with a poor control group than if the comparator receives strong support. In the trial of therapeutic communities designed to reduce recidivism in delinquents, the control group were in the ‘control’ house. However this control house had many defects and several of the staff were arrested for abusing the boys – hardly a typical control environment. But unless there is a good description of the comparator, any success or failure of the new intervention will be hard to interpret.

The 2001 CONSORT statement, in Item 4 describing the intervention states:

Authors should describe each intervention thoroughly, including control interventions. The characteristics of a placebo and the way in which it was disguised should also be reported. It is especially important to describe thoroughly the ‘usual care’ given to a control group or an intervention that is in fact a combination of interventions. (Moher, Schulz, & Altman, 2001)

Nevertheless, it was not clear how a control group should be described as reflected by Freedland et al. ‘s (2011) analysis of the variety of possibilities for describing a ‘usual care’ control group. Freedland et al. (2019) has also discussed the issues to consider when selecting as well as reporting control groups.

TIDieR emphasised the importance of specifying control groups content in as much detail as the experimental intervention but even then, detail about active content could be missed. Control groups vary enormously in the amount of active content they receive and seminal work by de Bruin and colleagues demonstrate how important it is to take that into account when interpreting the results of BCI trials. The amount of active behaviour change support given to control groups varies widely and when taken into account in meta-regression, has been shown to affect the results of the trials (de Bruin, Viechtbauer, Hospers, Schaalma, & Kok, 2009; Black et al., 2020). Control group support can explain differences in outcome not only between different interventions but also between different control groups (de Bruin et al., 2010; Black et al., 2020). It is now clear that the attention that is gradually being paid to the reporting of interventions needs to be applied equally to the control groups.

Having learned so much, it has become apparent that more needs to be done to ensure that BCIs can be delivered and reported in a manner which is replicable and which permits aggregation of evidence in a systematic and valid manner.

## What we have still to learn … 

Progress to date has exposed the deficits in previous BCI reports but additionally points to what more needs to be done. Decisions need to be made about which interventions should be investigated and how to optimise resources (Armitage et al., 2021; Collins et al., 2007). Surprisingly we continue to use as comparator control groups something akin to ‘treatment as usual’. This would be reasonable if absolutely no progress had been made. Whereas there is usually some evidence of effective BCIs and surely this is what any new intervention should be tested against. To some extent this may be what is already happening as improved methods become part of usual care, but, unless the control or comparator condition is well-described this will not be clear. If we were comparing a new intervention against a clearly specified comparator, then we might begin to use more Bayesian methods of analyses as we would be able to state the prior likelihood of the comparator being successful and could make considered estimates of the likely gains with the new BCI, rather than testing against a null hypothesis assuming no gain in the comparator group.

However, even with the best of BCT taxonomies and the best CONSORT, TIDieR and Cochrane reporting and synthesising, it is still the case that BCIs are incompletely reported and that the evidence used in designing BCIs and comparators is based on incomplete or dated evidence. The efforts required to reshape the vast quantity of BCI reports by getting additional information from authors and using taxonomies to conduct extensive annotations is barely feasible without using enormous resources, and even then the accumulation of evidence is likely to be too slow to be useful for practice and policy and too late to inform ongoing BCI design and evaluation.. It continues to be difficult to obtain the best synthesised evidence in a comprehensive and timely manner.

**Figure 1.**
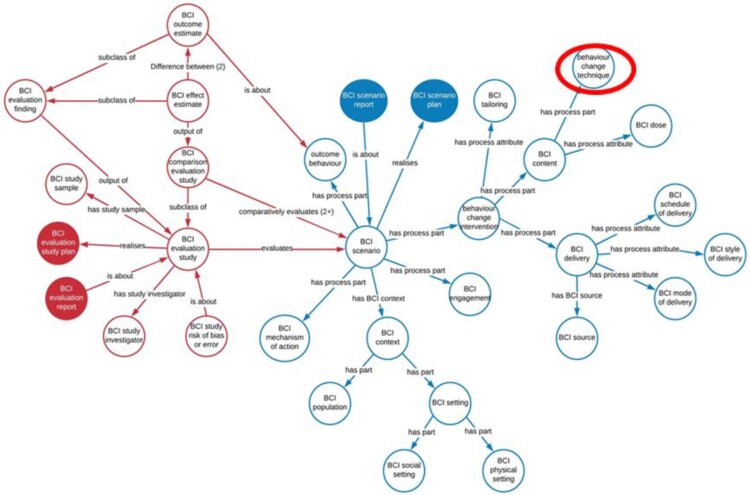
The Behaviour change Intervention Ontology Upper Level (Michie et al., 2021)

The Human Behaviour-Change Project aims to overcome some of these problems by developing a systematic method of finding published BCI reports, annotating the BCI content, synthesising themand interpreting the evidence – all implemented by computer, using machine learning and AI – and the results made accessible via a user-friendly user interface (Michie et al., 2017). The first step was the development of a BCI Ontology (BCIO) to represent the information contained in the reports. An ontology includes precise labels definitions of all the ‘entities’ included and goes beyond a simple hierarchy by allowing different kinds of relationship between entities and by ensuring interoperability with other ontologies (Michie & Johnston, 2017). The complexity of BCIs is illustrated in the upper level of the BCIO where BCTs define only one element of the BCI (see Figure 1) (Michie et al., 2021). A report of a BCI should also specify the ‘dose’ delivered and any tailoring to individuals as well as how it was delivered, when, how and by whom. The full BCI scenario also requires information about the context including the setting e.g. clinical or educational, and the population, e.g. age, health-status, socio-economic status. Each of these upper-level entities encompasses several lower levels. For example, the BCI setting has several layers of lower levels to describe where the intervention is delivered (Norris et al., 2020). BCTTv1 serves as the ontology for BCTs but requires further development of the BCTs, the clarity of their definitions and more logical organisation of the relationships between them to perform as an ontology. The full BCI scenario includes the evaluation study presented as an ontology of the entities needed to describe a competent evaluation such as a randomised controlled trial with information about risk of bias.

The BCI Ontology has several advantages over a simple hierarchy. First, the entities are precisely defined and so can be reliably identified in reports and used more widely by other researchers. Second, by using the BCIO to annotate many reports it is possible to build up a body of annotated reports which can then form a basis for machine learning of the annotations and so by-passing the need for the massive human effort of extracting key information from reports. Third, entities are linked by logical rather than empirical relationships; it is therefore possible to add additional entities as they are identified without revising the whole structure. By contrast, the consensus methods used to develop groupings of BCTs in BCTTv1 make it impossible to incorporate new BCTs without repeating the empirical consensus clustering procedures which would almost inevitable result in different groupings. In BCTTv1, the labels for the groupings were chosen to describe the content of each grouping whereas in an ontology, each BCT in a grouping would relate *logically* to the upper level label. Fourth, the hierarchical structure ensures that findings that are valid for the upper levels have validity for lower levels as the lower levels are contained within the upper levels; for example, a relationship that applies to ‘children’ also applies to ‘younger’ or ‘older’ children, as otherwise the relationship would not be applied at the upper level. Fifth, the BCIO has clear linkage to other related ontologies such as Cochrane’s PICO ontology (O’Connor, Green, & Higgins, 2008) which specifies population, intervention, comparison and outcome.

One challenge for the BCIO and for BCI research more generally is the problem of defining and classifying the target behaviour. Despite the study of behaviour being part of most definitions of psychology, the discipline has not developed a classification of behaviours and it has even been suggested that it is avoiding behaviour (Baumeister, Vohs, & Funder, 2007; Johnston & Dixon, 2008). Presseau et al. (2019) have proposed a minimum description of the behaviours as AACTT (action, actor, context, target, time). McEachan, Lawton, and Conner (2010) have proposed a classification based on repertory grid and focus group studies; and Nudelman and Shiloh (2015) report a taxonomy of behaviour clusters based on lay-people’s perceptions of similarities between behaviours. However, more work will be necessary to develop a satisfactory method of classifying behaviours beyond their use in specific domains and this is one of the challenges for the HBCP.

In order to make progress in defining what must be extracted from reports, West is developing methods of enhancing the reporting *per se* by developing a ‘Paper-Authoring-Tool (PAT)’ and so reducing the need for complex methods of finding the information in reports (West, 2020a) with initial detailed methods for addiction studies (West, 2020b). Finally, current and future progress toward open science is fundamental in making the evidence we report and synthesise available earlier and hopefully, more replicable (Nosek et al., 2015).

## Conclusions

Obviously, it would have been much better to have known way back then what is now widely known and has greatly improved the conduct and reporting of BCI evaluation studies. But it would be even worse if we had made no progress over the intervention years.

Major advances have been made in the design and reporting of BCI trials especially in the use of RCTs, better specification and measurement of outcomes, advances in statistical methods and evidence synthesis. At a somewhat slower pace has been the clarification of methods of designing and reporting the BCI *per se* but CONSORT and extensions, TIDieR and BCTs have all inched the field forward albeit rather slowly for control/comparator conditions. Much remains to be done in all of these areas and the HBCP and PAT work, while exciting and innovative, underlines how far we need to go to get optimal use of reported BCIs. Hopefully in a decade or so of further progress, it will be possible to reflect on how much better reporting and conduct of BCIs could have been in the early 2020s.

And all of this progress is important – for the advancement of the science of behaviour change, but also for addressing the accumulating social and health challenges that might profit from behaviour change solutions.

## References

[CIT0001] Abraham, C., & Michie, S. (2008). A taxonomy of behavior change techniques used in interventions. *Health Psychology*, *27*(3), 379–387. doi:10.1037/0278-6133.27.3.379 18624603

[CIT0002] Armitage, C. J., Conner, M., Prestwich, A., de Bruin, M., Johnston, M., Sniehotta, F. F., & Epton, T. (2021). Investigating which behaviour change techniques work for whom in which contexts delivered by what means: Proposal for an International collaboratory of centres for Understanding behaviour change (CUBiC). *British Journal of Health Psychology*, *26*, 1–14. doi:10.1111/bjhp.12479 33080120

[CIT0003] Baumeister, R. F., Vohs, K. D., & Funder, D. C. (2007). Psychology as the science of self-reports and finger movements: Whatever happened to actual behavior? *Perspectives on Psychological Science*, *2*(4), 396–403. doi:10.1111/j.1745-6916.2007.00051.x 26151975

[CIT0004] Begg, C., Cho, M., Eastwood, S., Horton, R., Moher, D., Olkin, I., … Stroup, D. F. (1996, Aug 28). Improving the quality of reporting of randomized controlled trials. The CONSORT Statement. *JAMA.*, *276*(8), 637–639. DOI:10.1001/jama.276.8.637 8773637

[CIT0005] Berli, C., Inauen, J., Stadler, G., Scholz, U., & Shrout, P. E. (2020). Understanding between-person interventions with time-intensive longitudinal outcome data: Longitudinal mediation analyses. *Annals of Behavioral Medicine*. doi:10.1093/abm/kaaa066/066/5901973. Epub-Ahead PMC8122473 32890399

[CIT0006] Black, N., Eisma, M. C., Viechtbauer, W., Johnston, M., West, R., Hartmann-Boyce, J., … de Bruin, M. (2020a). Variability and effectiveness of comparator group interventions in smoking cessation trials: A systematic review and meta-analysis. *Addiction*, *115*(9), 1607–1617. doi:10.1111/add.14969 32043675 PMC7496125

[CIT0007] Black, N., Johnston, M., Michie, S., Hartmann-Boyce, J., West, R., Viechtbauer, W., … de Bruin, M. (2020b). Behaviour change techniques associated with smoking cessation in intervention and comparator groups of randomized controlled trials: A systematic review and meta-regression. *Addiction*, *115*(11), 2008–2020. doi:10.1111/add.15056 32196796

[CIT0008] Bradley, C. (1993). Designing medical and educational intervention studies: A review of some alternatives to conventional randomized controlled trials. *Diabetes Care*, *16*(2), 509–518. doi:10.2337/diacare.16.2.509 8432226

[CIT0009] Bricca, A., Swithenbank, Z., Scott, N., Treweek, S., Johnston, M., Black, N., … de Bruin, M. (2021). Predictors of recruitment and retention in randomised controlled trials of behavioural smoking cessation interventions: A systematic review and meta-regression analysis. *Addiction*. 10.1111/add.15614 34159677

[CIT0010] Campbell, M., Fitzpatrick, R., Haines, A., Kinmonth, A. L., Sandercock, P., Spiegelhalter, D., & Tyrer, P. (2000). Framework for the design and evaluation of complex interventions to improve health. *BMJ*, *2000*(321), 694–696. doi:10.1136/bmj.321.7262.694 PMC1118564 10987780

[CIT0011] Campbell, M., Katikireddi, S. V., Hoffmann, T., Armstrong, R., Waters, E., & Craig, P. (2018). TIDieR-PHP: A reporting guideline for population health and policy interventions. *BMJ*, *361*. doi:10.1136/bmj.k1079 PMC5954974 29769210

[CIT0012] Chalmers, I., Dickersin, K., & Chalmers, T. C. (1992). Getting to grips with Archie cochrane's agenda. *BMJ*, *305*(6857), 786–788. doi:10.1136/bmj.305.6857.786. PMC 1883470. PMID 1422354. 1422354 PMC1883470

[CIT0013] Clarkson, J. E., Turner, S., Grimshaw, J. M., Ramsay, C. R., Johnston, M., Scott, A., … Pitts, N. B. (2008). Changing clinicians’ behavior: A randomized controlled trial of fees and education. *Journal of Dental Research*, *87*(7), 640–644. doi:10.1177/154405910808700701 18573983

[CIT0014] Collins, L. M., Murphy, S. A., & Strecher, V. (2007). The multiphase optimization strategy (MOST) and the sequential multiple assignment randomized trial (SMART): new methods for more potent eHealth interventions. *American Journal of Preventive Medicine*, *32*(5), S112–S118. doi:10.1016/j.amepre.2007.01.022 17466815 PMC2062525

[CIT0015] Cotterill, S., John, P., & Johnston, M. (2020). How can better monitoring, reporting and evaluation standards advance behavioural public policy? *Policy & Politics*, *V49*(1), 161–179. doi:10.1332/030557320X15955052119363

[CIT0016] Craig, P., Dieppe, P., Macintyre, S., Michie, S., Nazareth, I., & Petticrew, M. (2008). Developing and evaluating complex interventions: The new Medical Research Council guidance. *BMJ*, *337*. doi:10.1136/bmj.a1655 PMC2769032 18824488

[CIT0017] Davidson, K.W, Goldstein, M, Kaplan, R.M, Kaufmann, G.L, Orleans, C.T, & Whitlock, E.P. (2003). Evidence-based behavioural medicine: What is it and how do we achieve it. Annals of Behavioral Medicine, *26*(3), 161–171. 10.1207/S15324796ABM2603_01.161-171 14644692

[CIT0018] de Bruin, M., Viechtbauer, W., Hospers, H. J., Schaalma, H. P., & Kok, G. (2009). Standard care quality determines treatment outcomes in control groups of HAART-adherence intervention studies: Implications for the interpretation and comparison of intervention effects. *Health Psychology*, *28*(6), 668–674. doi:10.1037/a0015989 19916634

[CIT0019] de Bruin, M., Viechtbauer, W., Schaalma, H. P., Kok, G., Abraham, C., & Hospers, H. J. (2010). Standard care impact on effects of highly active antiretroviral therapy adherence interventions: A meta-analysis of randomized controlled trials. *Archives of Internal Medicine*, *170*(3), 240–250. doi:10.1001/archinternmed.2009.536 20142568

[CIT0020] Freedland, K. E., King, A. C., Ambrosius, W. T., Mayo-Wilson, E., Mohr, D. C., Czajkowski, S. M., … Riley, W. T. (2019). The selection of comparators for randomized controlled trials of health-related behavioral interventions: Recommendations of an NIH expert panel. *Journal of Clinical Epidemiology*, *110*, 74–81. doi:10.1016/j.jclinepi.2019.02.011 30826377 PMC6543841

[CIT0021] Freedland, K. E., Mohr, D. C., Davidson, K. W., & Schwartz, J. E. (2011). Usual and unusual care: Existing practice control groups in randomized controlled trials of behavioral interventions. *Psychosomatic Medicine*, *73*(4), 323–335. doi:10.1097/PSY.0b013e318218e1fb 21536837 PMC3091006

[CIT0022] Hardeman, W., Griffin, S., Johnston, M., Kinmonth, A. L., & Wareham, N. J. (2000). Interventions to prevent weight gain: A systematic review of psychological models and behaviour change methods. *International Journal of Obesity*, *24*(2), 131–143. doi:10.1038/sj.ijo.0801100 10702762

[CIT0023] Hoffmann, T. C., Erueti, C., & Glasziou, P. P. (2013). Poor description of non-pharmacological interventions: Analysis of consecutive sample of randomised trials. *BMJ*, *347*. doi:10.1136/bmj.f3755 PMC3768250 24021722

[CIT0024] Hoffmann, T. C., Glasziou, P. P., Boutron, I., Milne, R., Perera, R., Moher, D., … Michie, S. (2014). Better reporting of interventions: Template for intervention description and replication (TIDieR) checklist and guide. *BMJ*, *348*, g1687. doi:10.1136/bmj.g1687 24609605

[CIT0025] Hollis, S., & Campbell, F. (1999). What is meant by intention to treat analysis? Survey of published randomised controlled trials. *BMJ*, *319*(7211), 670–674. doi:10.1136/bmj.319.7211.670 10480822 PMC28218

[CIT0026] Johnston, M. (1976). Responsiveness of delinquents and Non-delinquents to social reinforcement. *British Journal of Social and Clinical Psychology*, *15*(1), 41–49. https://bpspsychub.onlinelibrary.wiley.com/doi/epdf/10.1111/j.2044-8260.1976.tb00005.x 10.1111/j.2044-8260.1976.tb00005.x 1260239

[CIT0027] Johnston, M. (1977). The treatment of agoraphobia in general practice. *Behavioural and Cognitive Psychotherapy*, *5*(4), 103–109. doi:10.1017/S2041348300015949

[CIT0028] Johnston, M. (1995). Health related behaviour change. In I. Sharpe (Ed.), *Cardiovascular Prevention in primary care: The Way forward. National forum for coronary disease Prevention* (pp. 37–47). London: Kings Fund.

[CIT0029] Johnston, M. (2014). Improving the reporting of behaviour change interventions. *The European Health Psychologist*, *16*(5), 181–189.

[CIT0030] Johnston, M., Bonetti, D., Joice, S., Pollard, B., Morrison, V., Francis, J. J., & MacWalter, R. (2007). Recovery from disability after stroke as a target for a behavioural intervention: Results of a randomized controlled trial. *Disability and Rehabilitation*, *29*(14), 1117–1127. doi:10.1080/03323310600950411 17612998

[CIT0031] Johnston, M., Carey, R. N., Connell Bohlen, L. E., Johnston, D. W., Rothman, A. J., de Bruin, M., … Michie, S. (2020). Development of an online tool for linking behavior change techniques and mechanisms of action based on triangulation of findings from literature synthesis and expert consensus. *Translational Behavioral Medicine*, doi:10.1093/tbm/ibaa050 PMC8158171 32749460

[CIT0032] Johnston, M., & Dixon, D. (2008). Current issues and new directions in psychology and health: What happened to behaviour in the decade of behaviour? *Psychology and Health*, *23*(5), 509–513. 10.1080/08870440701816728 25160717

[CIT0033] Johnston, M., Foulkes, J., Johnston, D. W., Pollard, B., & Gudmundsdottir, H. (1999). Impact on patients and partners of inpatient and extended cardiac counseling and rehabilitation: A controlled trial. *Psychosomatic Medicine*, *61*(2), 225–233. doi:10.1097/00006842-199903000-00015 10204976

[CIT0034] Johnston, M., & Lee-Jones, M. (1979). Evaluating post-surgical care in community hospitals. In D. J. Oborne, M. M. Gruneberg, & J. R. Eiser (Eds.), *Research in Psychology and medicine*. London: Academic Press. Vol II, 353–360

[CIT0035] Johnston, M., & Vögele, C. (1993). Benefits of psychological preparation for surgery: A meta-analysis. *Annals of Behavioral Medicine*, *15*(4), 245–256. doi:10.1093/ABM/15.4.245

[CIT0036] Kok, G., Gottlieb, N. H., Peters, G. J. Y., Mullen, P. D., Parcel, G. S., Ruiter, R. A., … Bartholomew, L. K. (2016). A taxonomy of behaviour change methods: An intervention mapping approach. *Health Psychology Review*, *10*(3), 297–312. doi:10.1080/17437199.2015.1077155 26262912 PMC4975080

[CIT0037] Loprinzi, P. D. (2016). Health behavior characteristics and all-cause mortality. *Preventive Medicine Reports*, *3*, 276–278. doi:10.1016/j.pmedr.2016.03.013 27419026 PMC4929208

[CIT0038] McCleary, N., Duncan, E. M., Stewart, F., & Francis, J. J. (2013). Active ingredients are reported more often for pharmacologic than non-pharmacologic interventions: An illustrative review of reporting practices in titles and abstracts. *Trials*, *14*(1), Article 146. doi:10.1186/1745-6215-14-146 23688143 PMC3663666

[CIT0039] McDowell, I. (2006). *Measuring health: A guide to rating scales and questionnaires* (3rd ed.). Oxford: Oxford University Press.

[CIT0040] McEachan, R. R., Lawton, R. J., & Conner, M. (2010). Classifying health-related behaviours: Exploring similarities and differences amongst behaviours. *British Journal of Health Psychology*, *15*(2), 347–366. Doi:10.1348/135910709X466487 19646330

[CIT0041] Michie, S., & Johnston, M. (2017). Optimising the value of the evidence generated in implementation science: The use of ontologies to address the challenges. *Implementation Science*, *12*, Article 131. doi:10.1186/s13012-017-0660-2 PMC5686802 29137660

[CIT0042] Michie, S., Johnston, M., & Carey, R. (2020). Behavior change techniques. In M. Gellman (Ed.), *Encyclopedia of behavioral medicine* (3rd ed). Springer. 10.1007/978-1-4614-6439-6_1661-2 (DOI is from 2016 entry

[CIT0043] Michie, S., Richardson, M., Johnston, M., Abraham, C., Francis, J., Hardeman, W., … Wood, C. E. (2013). The behavior change technique taxonomy (v1) of 93 hierarchically clustered techniques: Building an international consensus for the reporting of behavior change interventions. *Annals of Behavioral Medicine*, *46*(1), 81–95. doi:10.1007/s12160-013-9486-6 23512568

[CIT0044] Michie, S., Thomas, J., Johnston, M., Mac Aonghusa, P., Shawe-Taylor, J., Kelly, M. P., … West, R. (2017). The Human Behaviour-Change Project: harnessing the power of artificial intelligence and machine learning for evidence synthesis and interpretation. *Implementation Science*, *12*, 1–12. doi:10.1186/s13012-017-0641-5. Article 121. 29047393 PMC5648456

[CIT0045] Michie, S., West, R., Finnerty, A. N., Norris, E., Wright, A. J., Marques, M. M., … Hastings, J. (2021). Representation of behaviour change interventions and their evaluation: Development of the upper level of the behaviour change intervention ontology. [version2; peer review: 2 approved]. *Wellcome Open Research*, *5*, 123. 10.12688/wellcomeopenres.15902.2. 33614976 PMC7868854

[CIT0046] Michie, S., Wood, C. E., Johnston, M., Abraham, C., Francis, J. J., & Hardeman, W. (2015). Behaviour change techniques: The development and evaluation of a taxonomic method for reporting and describing behaviour change interventions (a suite of five studies involving consensus methods, randomised controlled trials and analysis of qualitative data. *Health Technol Assess (Rockv)*, *19*(99), 1–188. doi:10.3310/hta19990 PMC4781650 26616119

[CIT0047] Moher, D., Schulz, K. F., Altman, D. G., & CONSORT GROUP (Consolidated Standards of Reporting trials). (2001, Apr 17). The CONSORT statement: Revised recommendations for improving the quality of reports of parallel-group randomized trials. *Annals of Internal Medicine*, *134*(8), 657–662. doi:10.7326/0003-4819-134-8-200104170-00011 11304106

[CIT0048] Mokdad, A. H., Marks, J. S., Stroup, D. F., & Gerberding, J. L. (2004). Actual causes of death in the United States, 2000. *JAMA*, *291*(10), 1238–1245. doi:10.1001/jama.291.10.1238 15010446

[CIT0049] Montgomery, P., Grant, S., Mayo-Wilson, E., Macdonald, G., Michie, S., Hopewell, S., & Moher, D. (2018). Reporting randomised trials of social and psychological interventions: The CONSORT-SPI 2018 extension. *Trials*, *19*, 407. doi:10.1186/s13063-018-2733-1 30060754 PMC6066921

[CIT0050] Norris, E., Marques, M. M., Finnerty, A. N., Wright, A. J., West, R., Hastings, J., … Michie, S. (2020). Development of an intervention setting ontology for behaviour change: Specifying where interventions take place. *Wellcome Open Research*, *5*, 124. doi:10.12688/wellcomeopenres.15904.1 32964137 PMC7489274

[CIT0051] Nosek, B. A., Alter, G., Banks, G. C., Borsboom, D., Bowman, S. D., Breckler, S. J., … Yarkoni, T. (2015). Promoting an open research culture. *Science*, *348*(6242), 1422–1425. doi:10.1126/science.aab2374 26113702 PMC4550299

[CIT0052] Nudelman, G., & Shiloh, S. (2015). Mapping health behaviors: Constructing and validating a common-sense taxonomy of health behaviors. *Social Science & Medicine*, *146*, 1–10. doi:10.1016/j.socscimed.2015.10.004 26473449

[CIT0053] O’Connor, D., Green, S., & Higgins, J. P. T. (2008). Defining the review question and developing criteria for including studies. In J. P. T. Higgins & S. Green (Eds.), *Cochrane handbook for systematic reviews of interventions* (pp. 81–94). The Cochrane Collaboration. www.cochrane-handbook.org. 10.1002/9780470712184.ch5

[CIT0054] Page, M. J., McKenzie, J. E., Bossuyt, P. M., Boutron, I., Hoffmann, T. C., Mulrow, C. D., … Moher, D. (2021). Updating guidance for reporting systematic reviews: Development of the PRISMA 2020 statement. *Journal of Clinical Epidemiology*. doi:10.1016/j.jclinepi.2021.02.003 [Preferred Reporting Items for Systematic reviews and Meta-Analyses (PRISMA) statement] 33577987

[CIT0055] Perugini, M., Gallucci, M., & Costantini, G. (2018). A practical primer to power analysis for simple experimental designs. *International Review of Social Psychology*, *31*(1), doi:10.5334/irsp.181

[CIT0056] Peugh, J. L., Strotman, D., McGrady, M., Rausch, J., & Kashikar-Zuck, S. (2017). Beyond intent to treat (ITT): A complier average causal effect (CACE) estimation primer. *Journal of School Psychology*, *60*, 7–24. doi:10.1016/j.jsp.2015.12.006 28164801

[CIT0057] Pollard, B., Johnston, M., & Dieppe, P. (2006). What do osteoarthritis health outcome instruments measure? Impairment, activity limitation, or participation restriction? *The Journal of Rheumatology*, *33*(4), 757–763. Epub 2005 Dec 15. PMID: 16358368. 16358368

[CIT0058] Powell, R., Scott, N. W., Manyande, A., Bruce, J., Vögele, C., Byrne-Davis, L. M., … Johnston, M. (2016). Psychological preparation and postoperative outcomes for adults undergoing surgery under general anaesthesia. *Cochrane Database of Systematic Reviews*, (5). doi:10.1002/14651858.CD008646.pub2 PMC8687603 27228096

[CIT0059] Presseau, J., McCleary, N., Lorencatto, F., Patey, A. M., Grimshaw, J. M., & Francis, J. J. (2019). Action, actor, context, target, time (AACTT): a framework for specifying behaviour. *Implementation Science*, *14*(1), 102. doi:10.1186/s13012-019-0951-x 31806037 PMC6896730

[CIT0060] Shemilt, I., Simon, A., Hollands, G. J., Marteau, T. M., Ogilvie, D., O'Mara-Eves, A., … Thomas, J. (2014). Pinpointing needles in giant haystacks: Use of text mining to reduce impractical screening workload in extremely large scoping reviews. *Research Synthesis Methods*, *5*(1), 31–49. doi:10.1002/jrsm.1093. Epub 2013 Aug 23. 26054024

[CIT0061] Simpson, S., & Moore, L. (2021). ### *MRC guidance on complex interventions* {to be released spring 2021].

[CIT0062] Vieira, R., McDonald, S., Araújo-Soares, V., Sniehotta, F. F., & Henderson, R. (2017). Dynamic modelling of n-of-1 data: Powerful and flexible data analytics applied to individualised studies. *Health Psychology Review*, *11*(3), 222–234. doi:10.1080/17437199.2017.1343680 28629262

[CIT0063] West, R. (2020a). An online paper Authoring tool (PAT) to improve reporting of, and synthesis of evidence from, trials in behavioral sciences. *Health Psychology*, *39*(9), 846–850. doi:10.1037/hea0000927 32833486

[CIT0064] West, R. (2020b). Addiction paper authoring tool (PAT): a guide. *Qeios*. doi:10.32388/L2KF6W

[CIT0065] WHO. (2009). *Global health risks: Mortality and burden of disease attributable to selected major risks*. Geneva: World Health Organization. ISBN 978 92 4 156387 1. http://www.who.int/healthinfo/global_burden_disease/GlobalHealthRisks_report_full.pdf.

[CIT0066] Winkelstein, W. Jr (September 2009). The remarkable archie: Origins of the Cochrane collaboration. *Epidemiology*, *20*(5), 779. doi:10.1097/EDE.0b013e3181aff391. PMID 19680039. 19680039

